# Barriers and facilitators to accessing and utilising sexual and reproductive health services during the COVID-19 pandemic in Africa: a systematic review and meta-analysis

**DOI:** 10.1186/s12913-024-12028-2

**Published:** 2024-12-05

**Authors:** Obasanjo Afolabi Bolarinwa, Clifford Odimegwu, Kobi V. Ajayi, Tosin Olajide Oni, Rajeeb Kumar Sah, Akanni Akinyemi

**Affiliations:** 1 Department of Public Health, York St John University, London, United Kingdom; 2 Demography and Population Studies Programme, Schools of Public Health and Social Sciences, University of the Witwatersrand, Johannesburg, South Africa; 3 Department of Health Behavior, School of Public Health, Texas A&M University College Station, College Station, TX USA; 4 Department of Demography and Social Statistics, Obafemi Awolowo University, Ile-Ife, Osun State Nigeria; 5 School of Human and Health Sciences, University of Huddersfield, Queensgate, Huddersfield HD1 3DH United Kingdom

**Keywords:** Barriers, Facilitators, Access, Utilisation, Sexual and reproductive health services, COVID-19, Africa

## Abstract

**Background:**

Ensuring uninterrupted access and utilisation of sexual and reproductive health (SRH) services remains crucial for preventing adverse SRH outcomes. However, the unprecedented emergence of the 2019 coronavirus disease (COVID-19) significantly disrupted most of these services in Africa. Thus, we systematically reviewed and examined barriers and facilitators to accessing and utilising SRH services during the COVID-19 pandemic in Africa.

**Methods:**

We systematically searched five databases for relevant articles published between January 2020 to December 2022, and the articles were screened following the JBI and PRISMA guidelines. Meta-synthesis of barriers and facilitators to accessing and utilising SRH services during the COVID-19 pandemic were reported, while a meta-analysis of the pooled prevalence of barriers to accessing and utilising SRH services during the COVID-19 pandemic in Africa was analysed using R.

**Results:**

The pooled prevalence of barriers to accessing and utilising SRH services during the COVID-19 pandemic in Africa was 26%. Seven themes were developed for the identified barriers (disruption of healthcare services, fear and misinformation, limited availability of resources, place & region of residence, healthcare staff attitude/manpower, limited access to transportation, and stigma and discrimination), whilst six themes were developed for the identified facilitators (support for vulnerable populations, socio-demographic characteristics, community outreach programs, policy adaptations, telemedicine and digital health, and change in choice of sexual and reproductive commodities).

**Conclusion:**

This study found that the COVID-19 pandemic significantly impacted SRH service access and utilisation in Africa. We recommend that future research consider a longitudinal examination of the pandemic on African SRH services.

**Trial registration:**

PROSPERO registration number: CRD42022373335.

**Supplementary Information:**

The online version contains supplementary material available at 10.1186/s12913-024-12028-2.

## Background

Sexual and reproductive health (SRH) is essential for overall physical and mental well-being, and it underscores the essence of human rights and sustainable development [1]. In resource-constrained settings, like most parts of Africa, cascading problems and inadequate access to SRH services are major contributors to maternal, infant, and child mortality and morbidity. Limited access to SRH services in Africa is also linked to unintended pregnancies, unsafe abortion, sexually transmitted diseases (STI), gender-based violence, and other pregnancy and childbirth-related issues [1, 2]. Consequently, countries and regions of Africa account for a disproportionate burden of the consequences of adverse SRH services compared to other parts of the world. For example, sub-Saharan Africa is responsible for an estimated 70% of global maternal deaths and 52% of under-five child deaths [3, 4]. Despite actions to ameliorate the barriers to SRH services in Africa, projections towards attaining quality access and utilisation of SRH services seem to be stagnant, mostly due in part to the effect of the recent 2019 coronavirus disease (COVID-19) pandemic.

In the wake of the pandemic, SRH services such as family planning and contraceptive commodities, STI and HIV services, and abortion services were either partially or totally disrupted. Albeit an essential component of health, SRH services were considered non-essential in Africa and most of the world, thus eroding initial progress in addressing issues associated with the lack of these services. The consequences of these unprecedented disruptions have been well-documented, with devastating short and long-term implications for global health priorities [5–7]. Most notable is the fact that the COVID-19 pandemic diminished the chances of actualising the World Health Organisation (WHO)’s Triple Billion targets to improve the health of billions of people by 2023 and the United Nations (UN) sustainable development goals (SDGs) by 2030 [8]. More subtle, however, is the realisation that the pandemic exposed the inherent weakness in global health systems.

If the pandemic led to unexpected global health shortfalls even in countries with supposedly adequate health systems, what becomes the fate of countries and regions in Africa with already known strained health systems? Numerous reports allude to the fact that Africa bore and will continue to bear the brunt of the pandemic, including the pandemic’s after-effects due to various existing health system weaknesses. For example, chronic shortages in Africa’s healthcare workforce are substantive – only 3% of the global healthcare workforce is resident in Africa [9, 10]. In 2022, the WHO reported during the seventy-fifth World Health Assembly: Workforce 2030 [11] reiterated that exploding population growth combined with limited resources in Africa will shoulder a disproportionate weight of the global workforce shortages. An inadequate healthcare workforce manifests in terms of shortages (e.g., the number of healthcare workers), quality (e.g., the training of healthcare workers), and density (e.g., the number of healthcare workers per population) [12]. Indeed, the health workforce’s quantity, quality, and density are important indicators of health system strengthening, attaining key societal health indicators, and achieving optimal healthcare delivery and services. Relatedly, evidence indicates that the constellation of issues of Africa’s healthcare delivery and services are embedded within the WHO’s six building blocks of health systems [13]. The conglomeration of these issues will undoubtedly buffer the pandemic’s impact.

With an unpalatable health system in Africa, studies have reported the pandemic’s unequal impact across Africa, albeit mixed. At the COVID-19 outset, i.e., between March and April 2020, in South Africa, authors noted that the average monthly uptake of injectables (norethisterone enanthate and medroxyprogesterone acetate) declined by 45% in April 2020 compared with the monthly average two years prior. However, the uptake of oral contraceptive pills increased by 30% during the lockdown, whereas female sterilisation remained stable [14]. In Zambia, 31% of women using contraceptives remained steady before or after the pandemic. However, qualitative reports highlighted other nuanced barriers, including the lack of prioritisation of contraceptive services, transportation barriers, long facility queues, fear of contracting the virus, stock-out of injectables, and staff shortages [15]. Similar inconsistent findings related to access to and utilization of family planning counselling and services were reported in Nigeria [16], Ghana [17], Uganda [18], Malawi [19], and elsewhere in Africa [20, 21].

Furthermore, studies reported that STI/HIV testing, consultation, and treatment were also heavily impacted during the COVID-19 pandemic. Peters and colleagues analysed pre-exposure prophylaxis (PrEP) use among 546 adolescent girls and young women between March and April 2020 in South Africa. The study found that the number of visits for PrEP and HIV testing statistically declined by 29% while STI positivity tests increased statistically for Chlamydia trachomatis (23 to 30%), Neisseria gonorrhoeae (7 to 14%), and Trichomonas vaginalis (8 to 12%) [22]. Even among survivors of sexual violence in Kenya, the COVID-19 mitigation strategies upended their access to STI/HIV treatments. Ochieng (2022) reported that the proportion of rape survivors receiving the recommended HIV postexposure prophylaxis (PEP) and STI treatment decreased from 61 to 51% and 72 to 61%, respectively [23]. The findings underscore the ripple effect of the COVID-19 pandemic on an already vulnerable population sub-group, leading to devastating consequences. These trends of disrupted STI/HIV treatment and counselling have been recorded across Africa [24, 25], with one study projecting that compared with a one-year of no disruption to antiretroviral therapy drugs across half (50%) of the population living with HIV on treatment, there would be an estimated 1.63 times increase in HIV-related mortality over to a six-month interruption in sub-Saharan Africa alone [26]. Evidently, the adverse impact of the COVID-19 pandemic in Africa was not limited to family planning and STI/HIV treatments, but these unpleasant, although mixed, patterns were also reported among women seeking safe abortion services [14, 27, 28]. Suggesting that, on balance, SRH services in Africa were substantially impacted by the pandemic, and even though the pandemic is officially over, the lingering effects are yet to be fully known.

Despite burgeoning literature referencing the pandemic’s impact on SRH health services in Africa, what remains uncharacterised is the pandemic’s true effect on girls, women, and child health. Given the considerable variations in the determinants of SRH in Africa, ranging from individual and community to policy-level factors [29, 30], it is germane to investigate the pandemic’s impact on SRH services to inform and advance public health efforts, programs, and policies. Previous review studies are limited due to methodological rigour, limiting their generalisability [27, 31, 32]. This systematic review and meta-analysis will consider barriers to SRH services as associated factors/determinants that hinder or halt accessing and utilising required/needed SRH services during the COVID-19 pandemic in Africa, while facilitators to SRH services will be defined as associated factors/determinants that enables accessing and utilising SRH services during COVID-19 pandemic in Africa.

## Methods

### Study design

This study was conducted in line with the 2020 Joanna Briggs Institute (JBI) methodology guidelines for systematic review and meta-analysis [33, 34] and also followed the Preferred Reporting Items for Systematic Reviews and Meta-Analyses (PRISMA) reporting guidelines [35] in reporting relevant results. The combination of systematic review and meta-analysis in reviewing literature has been proven over time as an effective study design that gives a holistic view of health research and public health service delivery [36, 37]. This systematic review and meta-analysis was registered on the international Prospective Register for Systematic Reviews (PROSPERO) database, with protocol registration number CRD42022373335.

### Data source and search strategy

Two authors (OAB & KA) searched the African journals online (AJOL), PubMed, PsycINFO, CINAHL, and EMBASE databases for relevant articles published between January 2020 to December 2022. To ensure that the search terms and strategy were without bias, prior to the complete search in all the included databases, a preliminary search was conducted in PubMed and EMBASE to ensure that the text words or keywords were well scrutinised and matched the indexed keywords in the literature. The initial search showed substantial literature, and appropriate search terms were identified for subsequent searches. The initial preliminary search returned many relevant articles, indicating a thorough search; consequently, the same search terms were used in all other databases included in this study. All the search terms conducted in this study were in English (Table 1).

**Table 1:** Search terms

Category	Search Terms
**Barriers**	“barrier”, “barriers”
**Facilitation**	“facilitate”, “facilitated”, “facilitates”, “facilitating”, “facilitation”, “facilitative”, “facilitator”, “facilitators”
**Access**	“access”, “accessed”, “accessibility”, “accessible”, “accessing”
**Utilisation**	“utilization”, “utilisation”, “utilize”, “utilized”, “utilizing”, “utility”
**Sexual and Reproductive Health Services**	“family planning services”, “STIs”, “HIV testing”, “abortion services”, “sexual health”, “reproductive health”, “family planning services”
**COVID-19**	“covid 19”, “covid 19 pandemic”
**Geographical Location**	“africa”, “african”
**Date Range**	from 2020/1/1 to 2022/12/31

### Electronic database search

After the preliminary search in PubMed and EMBASE databases using relevant search terms and strategies, the same developed search terms were applied to other databases considered in this study, this includes the AJOL, PsycINFO, and CINAHL databases (Appendix I) to ensure consistency. The authors adopted another method to retrieve relevant articles for a comprehensive and extensive search by checking the reference list of eligible articles for additional relevant articles that met the study’s inclusion criteria. The concept of Population, Intervention, Context, Outcome, Timing and Study type (PICOTS) [38] was utilised to align with the study review research questions. Consequently, the PICOTS table was used to develop and review the inclusion and exclusion criteria (Supplementary Table 1).

### Eligibility criteria

#### Inclusion and exclusion criteria

The PICOTS table was applied to develop the inclusion and exclusion criteria in line with the study research question. According to the JBI [34], to minimise the risk of bias, the inclusion and exclusion criteria must be clearly stated from the initial review stage to ensure that relevant articles are included as eligible articles. The PICOTS strategy was utilised whilst searching and screening for eligible studies. Table 2 below shows the inclusion and exclusion criteria.

**Table 2:** The PICOTS, inclusion and exclusion criteria

PICOTS	Inclusion	Exclusion
Population	All populations	N/A
Intervention	Accessing and utilising sexual and reproductive health services during the COVID-19	- Access and utilisation of family planning counselling & services that did not consider the COVID-19 pandemic - Access and utilisation of STIs/HIV testing, consultation & treatments that did not consider the COVID-19 pandemic - Access and utilisation of abortion services that did not consider the COVID-19 pandemic
Context	Africa	- Studies that did not include countr(ies) in Africa.
Outcome	Barriers and facilitators	- Other focus outside barriers and facilitators
Timing	January 2020 to December 2022	- Before January 2020 - After December 2022
Study type	- Quantitative - Qualitative - Mixed method - English language	- Secondary review studies - Editorials and commentaries - Unpublished - Other languages

#### Study selection

The first stage of the study selection process was eliminating duplicates, titles, and abstract sifting. The initial returned articles were imported into EndNote X9 (Clarivate) to eliminate duplicates, titles, and abstract sifting. After this, the remaining articles were cross-checked against the developed inclusion and exclusion criteria to identify potentially relevant studies. To avoid missing important studies, all articles with little or no information in the abstract section were included for full-text reading. This process was completed by two authors (OAB and KA) and was cross-checked by a third author (TOO).

The second stage of the study selection process was the full-text screening. All the articles eligible for full-text screening after eliminating duplicates, titles and abstract sifting were downloaded for reading. In the case of full-text restricted access, the university library access was used to access the full text. OAB and KA were involved in the initial reading and selection of eligible articles, and TOO cross-checked this. Some full texts that required further reading were read more than once to ensure their suitability before inclusion or exclusion.

Detailed information about the study selection is provided in the result section of this study using the PRISMA flowchart. The selection of eligible studies was completed transparently by ensuring that all the processes involved in the study selection were well documented at every step, as Page et al. [39] recommended, and more information can be found in the study’s published protocol [40].

### Data extraction and synthesis of evidence

To ensure that no important information was left unextracted, the data extraction of relevant information was done twice based on specific details about the study of interest (Barriers and facilitators to accessing and utilising sexual and reproductive health services during the COVID-19 pandemic) [33, 41]. Two authors (OAB & KA) extracted the data in this study independently, which was reviewed by a third author (TOO). For the systematic review, all types of research studies were considered, and data such as authors’ name/ year of publication, study setting/country of publication, study design, population, size and target, COVID-19 period, identified barriers and facilitators to family planning counselling and services, STIs/HIV testing, consultation, and treatment, and provision of abortion services were extracted and reported. For the meta-analysis, only quantitative research studies were considered, and the reported prevalence rate of barriers to accessing and utilising SRH services with corresponding sample sizes for each eligible quantitative study was extracted. For SRH services, all data related to family planning counselling & services, STIs/HIV testing, consultation & treatments, and abortion services were extracted, and for barriers and facilitators, all data that showed associations to hindering or enabling access and utilisation to family planning counselling & services, STIs/HIV testing, consultation & treatments, and abortion services during COVID-19 pandemic in Africa.

### Quality assessment

According to Porritt [42], conducting a quality assessment of eligible articles for a review study is important to ensure that low-quality articles that may affect the review’s credibility are excluded. A quality assessment of 30 selected eligible articles was independently assessed by two authors (OAB & KA) using the mixed Methods Appraisal Tool (MMAT) [43] to ensure that all included studies were of all high-quality and where there is a discrepancy in the quality of the study to be included or excluded, the third author (TOO) was involved in resolving the disagreement. The MMAT is often utilised to evaluate and appraise quantitative, qualitative, or mixed-methods research designs. This tool assessed the eligible articles included in this review since all research study designs were considered [44]. The MMAT was used to evaluate and appraise the research designs, methodology, participant recruitment, data collection, the studies’ aims, data analysis, presentation of the findings, the discussion and conclusion of studies; however, the included eligible articles in this review were appraised based on six methodological quality criteria: research questions, rate of non-response, representativeness of the target population, research measurement, and how the research questions were analysed [44]. All the scores were calculated, and no eligible selected article was dropped because the lowest score was 80% [44], as shown in Appendix II.

### Data synthesis and analysis

#### Variables and indicators

The primary outcomes for this study were access and utilisation of SRH services during COVID-19 in Africa. The SRH services covered access and utilisation of services such as family planning counselling & services, STIs/HIV testing, consultation & treatments, and abortion services. The secondary outcomes considered in this study were barriers and facilitators. Any factors that hinder/halt the supplying, accessing and utilisation of all the considered SRH services in this study were considered barriers, while factors or determinate that promote/enable the supplying, accessing and utilisation of all the considered SRH services were considered facilitators.

#### Systematic review

An emerging cluster approach was utilised to synthesise and aggregate identified barriers and facilitators to accessing and utilising SRH services during the COVID-19 pandemic in Africa from the included eligible studies [45–47]. This was done in two phases. Firstly, we created a manifest content analysis to identify the barriers and facilitators as reported in the eligible included studies. This was done by considering the reported associated variables that created barriers or facilitated access to and utilisation of SRH services during the COVID-19 pandemic in Africa. The second stage was to structure and sort those identified barriers and facilitators into clusters; this involves the clustering of all the barriers and facilitators in each eligible included study according to how they relate to one another, which led to the identification of themes and sub-themes for barriers and facilitators [46]. The development of the clusters was achieved through in-depth reading and interpretation of the included articles’ results section by (OAB & KA). All eligible studies were synthesised, regardless of their research design. The developed themes were identified and organised using MS Word and Excel.

#### Meta-analysis and funnel plot

The reported effect sizes (proportions) in the 13 quantitative studies that reported the prevalence of barriers to SRH services during COVID-19 in Africa were logit-transformed to prevent overestimating their precision. This was performed before estimating the pooled prevalence of barriers to SRH services because proportions are restricted between 0 and 1, which could artificially compress their standard errors. After the logit transformation, the raw proportions deviated further from the initially distorted ‘normal’ distribution pattern (W = 0.73 at *p* < 0.05 and W = 0.81 at *p* < 0.05, respectively). The Knapp-Hartung adjustment for the random effects model was then applied by specifying the *‘method.tau’* argument as “PM” in the *update.meta()* function of *dmetar package* in R [48, 49]. Based on t-distribution, the Knapp-Hartung adjustment was used to test if the pooled effect size is significant. At *p* < 0.001, the test shows that the pooled effect size is significant.

The funnel plot was illustrated as a scatter plot of the observed effect sizes (expressed as the standardised mean difference) on the x-axis against the measure of their standard errors on the y-axis. A vertical line in the middle represents an average effect size for the funnel. Should there be no small study effects, the studies are expected to follow the idealised funnel shape illustrated in the diagram. In the absence of publication bias, the data points on the plot form an upside-down, roughly symmetrical funnel. Studies with low standard errors thus should lie in the plot’s top part and close to the pooled effect size [48, 49].

On the other hand, studies with increasing standard errors open up the plot (found at the base of the funnel) and are more scattered to the right and left of the pooled effect. In other words, the low effect size of the studies at the base of the plot has low precision and is, therefore, likely not reflective of the actual effect on the population. The contour-enhanced option *col.contour* of the *funnel.meta()* function with significance thresholds at 0.99, 0.95 and 0.0 (equivalent to *p*-value at 0.01, 0.05, 0.1) was applied to the studies to prevent a wrong attribution of publication bias to the asymmetric pattern of the funnel plot [50, 51]. All the analyses were performed by TOO & OAB using R (R Core Team).

## Results

**Fig. 1:**
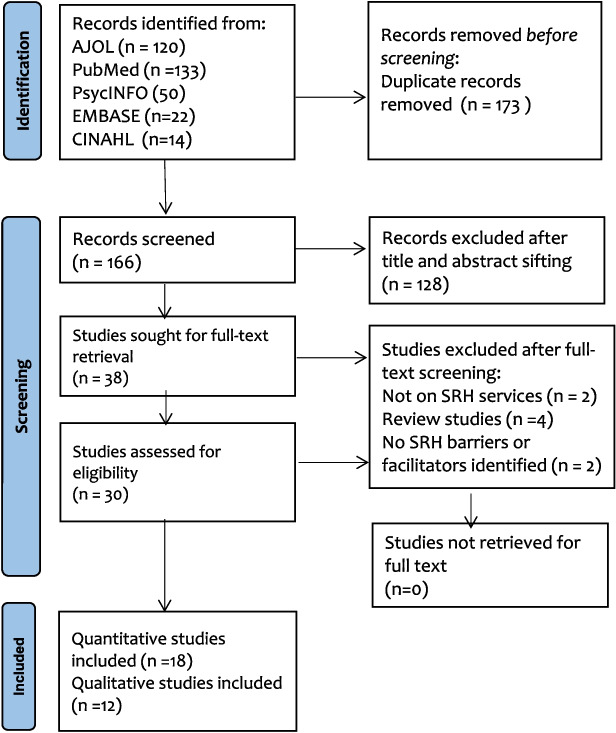
PRISMA 2020 Flow diagram [39]

From the 339 records obtained from the initial search of the five included databases, 173 duplicates were removed, whilst 166 were included for title and abstract screening. One hundred twenty-eight (128) studies were removed after the title and abstract screening because the studies did not meet the inclusion criteria. Of 38 studies included for full-text screening, 8 studies were excluded because the studies did not meet the inclusion criteria; 2 out of the 8 studies did not report results on access or utilisation of any SRH services considered in this study, 4 studies were review studies, and 2 studies did not report any form of barriers or facilitators on access and utilisation of SRH services. The final studies included in this systematic review and meta-analysis were 30; 18 studies utilised quantitative research [16, 19, 52–67], whilst 12 studies utilised qualitative research design [17, 21, 68–77]. Full details are presented in the PRISMA flow diagram (Fig. 1). Information on excluded studies can be found in Appendix III.

### Systematic review results

#### Characteristics of included studies and key findings

Six of the 30 eligible included studies were conducted in multiple African countries [19, 21, 60, 65, 68]. A study each from Ghana [78], the Democratic Republic of Congo [69], Burkina Faso [54], and Mali [70]. Four studies from South Africa [52, 57, 66, 67], and three studies were conducted each in both Kenya [53, 71, 73] and Nigeria [16, 55, 76]. Five studies were conducted in Ethiopia [59, 62–64, 72]. Most of the studies were conducted between 2020 and 2021. Ten studies focused only on women’s SRH services [19, 54, 55, 57, 58, 60, 62, 64, 65, 68], whilst 6 studies focused on young adults (male & female) and adolescent girls & young women only [21, 53, 61, 72, 74, 75]. Major reported results on barriers to access and utilisation of sexual and reproductive health services during COVID-19 in Africa were healthcare services disruptions, fear of misinformation and limited availability of alternative resources, whilst the facilitators were majorly adoption of multiple community outreach programmes and use of telemedicine/social media medium for consultations (Supplementary Table 1). The included studies’ geographical distribution is shown in Appendix IV.

### Meta-synthesis

Synthesised contents were grouped into 7 themes with 18 sub-themes for barriers to family planning services, 7 sub-themes for barriers to STIs/HIV-related services and 8 sub-themes were developed for barriers to abortion services. The seven themes developed for barriers to access and utilisation of SRH services during the COVID-19 pandemic in Africa include disruption of healthcare services, fear and misinformation, limited availability of resources, place & region of residence, healthcare Staff attitude/Manpower, Limited access to transportation, and stigma and discrimination from all the eligible included studies [16, 17, 19, 21, 52–77]. More than half of the eligible studies reported disruption of healthcare services as a major barrier for not accessing or utilising sexual and reproductive health services during the COVID-19 pandemic in Africa, whilst the least reported barrier was stigma and discrimination (Table 3).

**Table 3:** Barriers to access and utilisation of sexual and reproductive services during COVID-19 pandemic in Africa

Studies	Themes	Family planning counselling and services	STIs/HIV testing, consultation, and treatment	Provision of abortion services
Bercu et al., 2022 [68]; Bolarinwa, 2021 [31]; Brunie et al., 2022 [19]; Carter et al., 2022 [69]; Druetz et al., 2022 [54]; Egwuatu et al., 2022 [55]; Endler et al., 2021 [56]; Fuseini et al., 2022 [58]; Gebreegziabher et al., 2022 [59]; Haidara et al., 2022 [70]; Hassan et al., 2022 [71]; Karp et al., 2021 [60]; Mambo et al., 2022 [61]; Mavodza et al., 2022 [74]; Michael et al., 2021 [16]; Olugbade et al., 2022 [76]; Shuka et al., 2021; Tiew et al., 2022 [77]; Tilahun et al., 2022 [64]; Wood et al., 2021 [65]; Pillay et al., 2021 [66]; Dorward et al., 2021 [67]	Disruption of healthcare services	- Limited access to condoms due to change in priority of healthcare service - Lack of availability of preferred contraception methods due to service shutdown was linked with non-use and unintended pregnancies - Shortage of sexual and reproductive health services, such as family planning, linked to increased pregnancies. - Low enrolment in family planning programmes - Women in Kenya and Burkina Faso were not using any contraception method due to COVID-19 service disruption. - Locked drugs/pharmacies restricted access.	- Limited access to Testing and treatment services for sexually transmitted infections - Access to HIV/AIDS services was also limited - Decrease in HIV testing and ART collection	- Challenges in seeking self-abortion Services - Locating a private & comfortable place - Self-reported history of abortions in the previous 12 months increased due to limited access to healthcare services for abortion. - Lack of access to surgical and medical abortion. - Safe abortion care significantly decreased.
Biney et al., 2022 [78]; Haidara et al., 2022 [70]; Jones et al., 2022 [72]; Malkin et al., 2022 [21]; Michael et al., 2021 [16]; Molla et al., 2021	Fear and misinformation	- Reduction in the patronage of family planning services due to fear of contracting the virus in healthcare centres - Fear of visiting healthcare centres due to COVID-19	- Reduction in STI testing due to fear of contracting the virus in healthcare centres - Youths living with HIV and commercial sex workers could not visit healthcare due to the fear of contracting the virus.	-
Egwuatu et al., 2022 [55]; Haidara et al., 2022 [70]; Hassan et al., 2022 [71]; Mavodza et al., 2022 [74]; Tiew et al., 2022 [77]	Limited availability of resources	- Demand for family planning services was reduced due to cost barriers - Women’s financial insecurity and dependence on partners or parents impacted women’s choice of using family planning - Youths faced challenges using family planning services due to worsening socioeconomic status		- During the pandemic, participants were more likely to encounter financial and housing complications to self-manage abortion than before the pandemic.
Congo et al., 2022 [53]; Michael et al., 2021 [16]	Place & region of residence	- The risk of pregnancy was higher in certain regions, such as Nairobi and Thika, during the lockdown than in pre-COVID-19 lockdown due to limited access to contraceptive use. - The Rural resident has limited access to family planning use.		
Haidara et al., 2022 [70]; Long et al., 2022 [73]; Meyer et al., 2022 [75]	Healthcare Staff attitude/Manpower	- Shortage of family planning supplies due to staffing issues - Healthcare worker strike impacted family planning program implementation - Adolescents and youths reported significant difficulties in accessing due to stigmatization among service providers	- Healthcare worker strikes impacted STI program implementation	-
Hassan et al., 2022 [71]; Mambo et al., 2022 [61]; Michael et al., 2021 [16]; Tiew et al., 2022 [77]	Limited access to transportation	- Movement restriction limited access to transportation, which affected social networks and access to friends to encourage family planning use. - Long distance to family planning facilities	- Long distance to STIs testing & treatment centres & limited availability of transport services	- Long distance to abortion centres & limited availability of transport services - Lack of supportive infrastructures such as transportation to get to facilities for abortion services
Meyer et al., 2022 [75]	Stigma and discrimination	- Biases and judgemental attitudes towards accessing and using family planning services were persistent.	-	

Synthesised contents were grouped into six themes with 9 sub-themes for facilitators/enablers of family planning services, 3 sub-themes for facilitators of STIs/HIV-related services and 2 sub-themes were developed for facilitators of abortion services. The six themes developed for facilitators to access and utilisation of SRH services during the COVID-19 pandemic in Africa were support for vulnerable populations, socio-demographic characteristics, community outreach programs, policy adaptations, telemedicine and digital health, and change in choice of sexual and reproductive commodities from 10 eligible included studies [21, 52, 53, 56–58, 60, 68, 70, 73]. Family planning services enablers during COVID-19 within the sub-theme include the area of residence, wealth status, distribution of condoms within the community, digital interventions, including phone-based services, shift and change from short-acting contraceptive to long-acting contraceptive methods, facilitators to STIs/HIV testing and treatments include the adaptation of community services by healthcare providers, phone-based services and telemedicine digital interventions whilst the provision of abortion services were facilitated by supports of accompaniment, self-managed abortion, change in abortion policies in countries with mild restriction towards abortion services, and telemedicine consultation to women who needed abortion services studies [21, 52, 53, 56–58, 60, 68, 70, 73] Table 4.

**Table 4:** Facilitators to access and utilisation of sexual and reproductive services during the COVID-19 pandemic in Africa

Studies	Themes	Family planning counselling and services	STIs/HIV testing, consultation, and treatment	Provision of abortion services
Bercu et al., 2022 [68]	Support for vulnerable populations			Accompaniment supports to help in self-managed abortion.
Bolarinwa, 2021 [31]	Socio-demographic characteristics	- Race/ethnicity, wealth status, and residential area predicted condom access.		
Congo et al., 2022 [53]; Haidara et al., 2022 [70]; Malkin et al., 2022 [21]	Community outreach programs	- Condom shared within the community was associated with a 71% reduced hazard of pregnancy. - Healthcare providers made adaptations for community services on family planning use. - Current programmes changed to the community service model	- Healthcare providers made adaptations for community services on STI services	
Endler et al., 2021 [56]	Policy adaptations			- Countries with mildly restrictive abortion policies implemented COVID-19-related changes to aid continued access to abortion services compared to none among countries with severe restrictions.
Endler et al., 2022 [57]; Haidara et al., 2022 [70]; Long et al., 2022 [73]; Malkin et al., 2022 [21]	Telemedicine and digital health	- Phone-based services by healthcare workers on the family planning use - Digital interventions increased the enrolment of new family planning users - The Mhuri/Imuli project adapted its mobile outreach model to upscale the use of family planning	- Phone-based services by healthcare workers on STI testing - Digital interventions increased the testing of STIs among new clients	- Women in the telemedicine group had a complete abortion compared to women in the standard care group
Fuseini et al., 2022 [58]; Karp et al., 2021 [60]; Malkin et al., 2022 [21]	Change in choice of sexual and reproductive commodities	- A significant shift to long-acting reversible contraceptives (LARC) methods - Change in choice of contraception to more lasting methods		-

### Meta-analysis results

#### Pooled prevalence

**Fig. 2:**
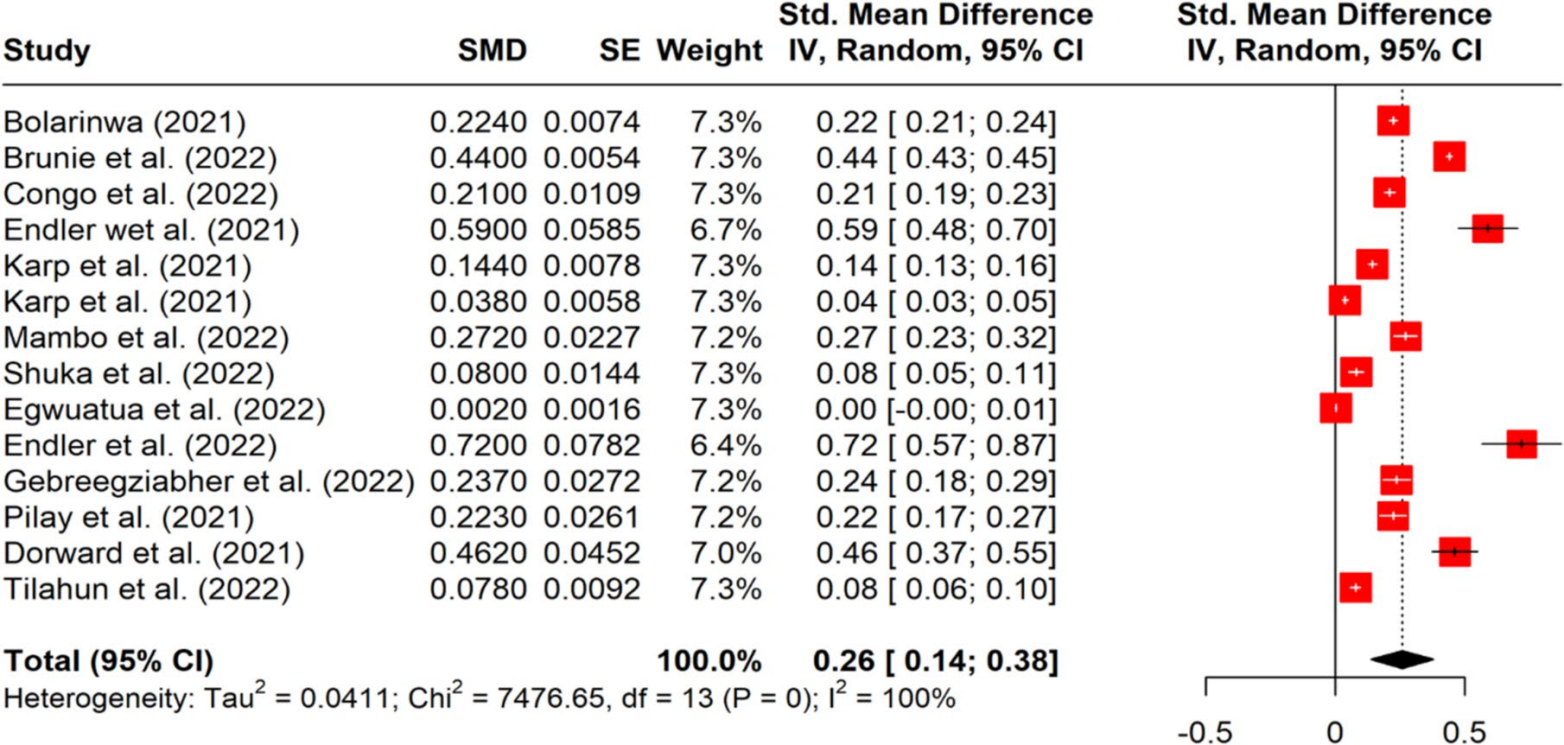
Pooled prevalence of barriers to access and utilisation of sexual and reproductive health services during COVID-19 in Africa

The results of the pooled prevalence of barriers to SRH services are presented in Fig. 2. The ‘SE Weight’ column shows the percent prevalence of each study used in the random-effects model (restricted maximum likelihood estimator). As shown in the figure, the greatest weight (%) in the meta-analysis was 7.3% from eight of the thirteen studies. The lowest weight is 6.4% and was from the study by Endler et al. [57]. This is noticeable because the confidence interval (CI) around the study’s pooled effect is the largest (0.57–0.87), thus making its effect size estimate the least precise of all. The pooled effect size is g ≈ 0.26 (overall prevalence of barriers), and its 95% CI ranges from g ≈ 0.14 to 0.38.

However, the analysis shows that the pooled effect from the random-effect model, which gave 0.26, deviated considerably from the fixed-effect model result, which was 0.225. The adjusted pooled effect was 0.259, thereby confirming the significance of the reported pooled effect at 26%. The between-study heterogeneity test shows that τ2 = 0.0411 (0.0213; 0.1157). Given that the confidence interval of τ2 does not include zero, tau2 at 0.0411 is thus significantly greater than zero, indicating that between-study heterogeneity exists in the data used for the meta-analysis; hence, the random-effects model was a good choice.

### Funnel plot

A funnel plot of the 13 quantitative studies that reported the prevalence of barriers to SRH services during COVID-19 in Africa is displayed in supplementary Fig. 1. Six of the 13 studies had high precision (< 0.4 standard errors) and followed the idealized funnel pattern, concentrating on the estimated true effect. However, 1 study had very low effect sizes and was outside the funnel.

As shown in the three shaded regions, 10 of the 14 studies have significant effect sizes at *p* < 0.01 and *p* < 0.05. Three of the 4 studies that were not found significant had low standard errors, even though they were not within the idealized funnel. Therefore, Egger’s regression test was conducted to objectively and cautiously interpret the funnel plot’s asymmetry. The intercept of the regression was β = 16.3. This intercept is significantly greater than zero (t = 2.296; *p* = 0.05) and only shows that the funnel plot data is truly asymmetrical. Further, a test of Egger’s intercept was conducted by assigning the “linreg” option to the “method.bias” argument of the *metabias()* function. The test shows the significance of the intercept at t=−2.57; *p* < 0.05.

## Discussion

This study investigated the barriers and facilitators to SRH services such as family planning, STI/HIV treatment, and abortion services in Africa during the COVID-19 pandemic. Consistent with the literature, although mixed, we found substantial disruption to access and utilization of SRH services. Most notably and based on data from 14 articles, we found a 26% overall pooled prevalence of barriers to SRH services during the study period, suggesting a substantial number of people living in Africa had limited or inadequate care related to their sexual health or reproductive health. The meta-analysis finding substantiates the thematic synthesis, revealing numerous barriers and facilitators ranging from proximal to distal factors that complicate and facilitate the utilization and access to timely, safe, and quality SRH care during the COVID-19 pandemic. This study found that despite the challenges faced by health systems, governments, and other stakeholders of some countries in Africa, some African countries found and leveraged innovative and collaborative approaches to ensure patients continued to access SRH related-care. The results demonstrate that despite adversity and amid public health emergencies in resource-constraints regions, SRH care can still be prioritized and protected given its critical role in the overall health and well-being of all, regardless of sex and gender.

Concerning the barriers to SRH services in Africa, we found that they were clustered around the disruption of healthcare services, fear and misinformation, limited availability of resources, place and region of residence, healthcare staff attitude/manpower, limited access to transportation, and stigma and discrimination. These findings parallel results on SRH and other health conditions from the United States [79] and the United Kingdom [80], with more coordinated health systems compared with Africa. Despite the heterogeneity in economic capabilities and health system strength across Africa, it appears that the total lack of or limited SRH commodities was mostly reported in the literature and may have the greatest impact in attaining the WHO’s Triple Billion and UN sustainable development goals [8]. Even though the barriers found in this study have varying consequences on access to SRH, it is important to understand why most of the barriers reported were clustered around the disruption of healthcare services. Firstly, and as mentioned earlier, Africa has a strain healthcare system, low physician quantity, quality, and density, and poor budgetary healthcare allocation [11–13]. In addition, despite the existing and long-standing healthcare system issues in Africa, at the outset of the pandemic, the region was sidelined by pharmaceutical companies in the global north and strict regulatory policies instituted by high-income countries in equitably distributing COVID-19 supplies [81]. Parts of the region experienced harsh and unwarranted economic restrictions and penalization for discovering new COVID-19 mutations [82, 83], and in some cases, the efforts to innovate and localize COVID-19 preventative measures (e.g., COVID-19 vaccines) were stifled, and some countries in Africa openly rejected these preventive measures [84–86]. These unjust and preventable systemic mistreatments and injustices undergirded by neocolonialism [87] exacerbated the pandemic’s negative effect and disproportionately impacted the SRH of Africans. Indeed, the conflation of these factors is a recipe for overall adverse health outcomes and quality of life for Africans, especially young girls and women and vulnerable populations [88, 89].

Despite the effect of the pandemic on the access and utilization of SRH services in Africa, some countries were swift in adjusting and adapting to the challenges brought by the pandemic to ensure that they continued to deliver quality healthcare through several mechanisms ranging from system-level changes, such as policy reforms and the use of telehealth and other forms of digital health to accelerate and augment healthcare to a more hands-on bottom-approach such as the use of community workers and partners [90, 91]. While these actions are laudable, it reinforces the need for a proactive versus reactive approach to healthcare even beyond the pandemic. For example, telehealth was emergent or nonexistent before the pandemic in some African countries, particularly for SRH care [92]. Interestingly, the pandemic catalysed unprecedented acceptance and a shift in operation to allow more innovation in delivering SRH care.

Similar to our study’s findings, a scoping review conducted by Doraiswamy et al. [93] reported that Asian countries swiftly adopted telehealth in delivering healthcare services, ultimately improving the overall healthcare services delivery. Nevertheless, Ftouni et al. [94] noted that healthcare services delivery through telehealth/telemedicine may pose some challenges around privacy and infrastructural deficit; however, it’s important to note that the use of telehealth during COVID-19 has contributed immensely to healthcare access and services delivery more than the challenges highlighted, especially around the delivery of SRH access and services [95, 96].

Although the WHO has declared that the pandemic is over, the rippling effects still linger [97, 98]. The results from this study are a reminder that when systems and infrastructures are not put in place to withstand new or recurrent public health emergencies, it can lead to devastating consequences that, in some cases, may be preventable, as in the case of SRH care. Our analysis revealed that for Africa to continue to promote and protect girls’ and women’s health, she must be willing to accept innovation, sustain the COVID-19 response momentum, evaluate and re-evaluate stifling and archaic policies, open to multisectoral partnerships, and most importantly be willing to accept change.

### Strengths and limitations

This study adds to the literature by providing critical insights into the pandemic’s impact on African SRH services. Using a meta-analysis bolstered this study’s robustness and statistical power by reporting generalizable estimates to extrapolate to the countries not included in the analysis. These strengths notwithstanding, there are a few limitations that must be discussed. Firstly, despite our best efforts to ensure we captured all the African countries in our search strategy, only fifteen countries were represented in this study. As a result, we may be unable to precisely infer that our findings apply to the other African countries not represented in this study. This is especially true when considering the heterogeneity of the African populace. However, because this study’s findings are parallel to those reported worldwide, we believed that the results from this study could be meaningful across Africa. Another noteworthy limitation is that we restricted our study to when COVID-19 had pandemic status. It is possible that more studies may have been published afterwards that might provide additional insights, particularly as it relates to the lingering effects of the pandemic.

Similarly, our restrictions did not allow us to conduct a comparison analysis during and after the COVID-19 pandemic status to identify, if any, patterns of SRH access and utilization across Africa. Lastly, our study acknowledges that given the heterogeneity of the African nation, it would have been important to stratify our findings by region and/or country to uncover nuances in the barriers and facilitators to SRH services, with region-and country-specific implications. Still, this study’s result is crucial to understanding the impact of the pandemic in Africa and will be most certainly useful in future pandemics since the question is not about if but when another public health emergency will emerge.

### Implication for sexual and reproductive health rights and policies

This study has several implications for SRH rights and policies in the African context. Firstly, our study spotlights the need for robust policies that consider all rights, regardless of socioeconomic and health status. By focusing on health policy reform, including expanding budgetary allocation to healthcare – a persistent issue in the region, increasing the quantity and quality of the healthcare workforce, and creating an ecosystem that allows for a continuous feedback loop and evaluation of the effects (including the unintended consequences) of policies, can strengthen the healthcare sector to be proactive in delivering SRH and overall health. Secondly, community health workers must be acknowledged as integral to delivering health services. Research shows that community health workers can augment care and increase healthcare adherence at the community level because they are part of the community wherein they work [99]. They also can build community partnerships which can help strengthen relationships between the community and the hospital. Thus, this study calls for a more concerted effort by policymakers and health systems to collaboratively prioritise and include community workers in the delivery of SRH services. Thirdly, there’s a need for innovation in the health systems in Africa.

Notwithstanding the problems (e.g., data breaches, financial costs, implementation of digital health infrastructure, availability, accessibility, and technical know-how of digital health from the patient and provider) associated with the uptake of digital health care in resource-constrained regions, abundant literature has shown that the benefits far outweigh the risks [92], particularly when the preventive measures to protect patients are put in place. The benefits for SRH in light of digital health include but are not limited to (1) uninterrupted healthcare even for remote or rural areas, (2) healthcare linkages with diverse care teams, (3) safe and confidential care, thereby removing the stigma and discrimination that may be associated with seeking SRH care, and (4) the ability to reach wider patient populations. One implication is that even in resource-constrained settings, digital healthcare does not compromise the quality of care. In addition, our study calls for evidence-based public health messaging. In our current clime, mis[dis]information is a rapid public health concern, which can lead to poor health choices and outcomes. Strategic messages in simple and plain language that patients can easily digest using diverse mediums such as billboards, flyers, mass media and social media platforms must be adopted and utilized nationally and locally. Because messaging goes beyond mass media or public messaging, improving patient-provider communication languages and encounters may mitigate distrust and misinformation.

#### Conclusion and recommendations for future studies

This study found that the COVID-19 pandemic significantly impacted SRH service access and utilisation in Africa during the pandemic. Albeit the findings parallel the literature conducted in other parts of the world, they present opportunities for future studies. Foremost, we recommend that future research should consider a longitudinal examination of the pandemic on SRH to understand and observe changes and patterns of the pandemic on SRH. This will be especially worthwhile when considering the pandemic’s ripple effect, such as long-haul COVID-19. Next, our study indicated that several programs and interventions were developed and implemented due to the pandemic. As such, future studies should evaluate SRH programs, interventions, and policies developed during the pandemic to ascertain such programs’ effectiveness, feasibility, and replicability in other settings. Such studies are critical, especially given the limited nature of health funding mechanisms – with a substantial stream of international donor funding in the region. Beyond the benefits of evaluating programs and policies for funding reasons, continuous evaluation may also help to know when and how to pivot from programs that may be unsustainable. Lastly, future quantitative and qualitative research should be conducted across other parts of the region to expand insights about country-specific factors affecting SRH services and health in general. Such knowledge will help formulate localized policies and programs that appeal to the needs of communities.

## Supplementary Information

Supplementary Material 1.

Supplementary Material 2.

## Data Availability

No datasets were generated or analysed during the current study.

## Abbreviations

SRH: Sexual and reproductive health
STIs: Sexually transmitted diseases
COVID-19: 2019 coronavirus disease
WHO: World Health Organization
UN: United Nations
SDGs: Sustainable development goals
PrEP: Pre-exposure prophylaxis
PEP: Post-exposure prophylaxis
JBI: Joanna Briggs Institute
PRISMA: Preferred Reporting Items for Systematic Reviews and Meta-Analyses
PROSPERO: Prospective Register for Systematic Reviews
AJOL: African journals online
PICOTS: Population, Intervention, Context, Outcome, Timing and Study type
MMAT: Mixed Methods Appraisal Tool
CI: Confidence interval
