# Supplementary material for: Barriers and facilitators to accessing and utilising sexual and reproductive health services during the COVID-19 pandemic in Africa: a systematic review and meta-analysis
Source: BMC Health Serv Res. 2024 Dec 5;24:1554. doi: 10.1186/s12913-024-12028-2 (PMC11622569; doi:10.1186/s12913-024-12028-2)
Supplement: Supplementary file 1 — Supplementary Material 1. [file 12913_2024_12028_MOESM1_ESM.docx]

**Appendix II****I: Details of the excluded studies and reasons for exclusion**

| **S/N** | **First Author’s surname (year)** | **Reason for exclusion** | **Flow diagram denoted** |
| --- | --- | --- | --- |
| 1 | Maithufi (2021) | The study outcome did not meet the eligibility criteria because the study did not cover SRH services | Not on SRH services during COVID-19 |
| 2 | Briggs (2020) | The study outcome did not meet the eligibility criteria because the study did not cover SRH services | Not on SRH services during COVID-19 |
| 3 | Bassey (2021) | This study was a review of findings on HIV Prevention and Treatment During the COVID-19 Pandemic in Nigeria, however, previously published review study was not stated as an inclusion criteria in the current study | Review studies |
| 4 | Esievoadje (2022) | This study was a review of findings on accessibility and utilization of family planning services in Nigeria, however, previously published review study was not stated as an inclusion criteria in the current study | Review studies |
| 5 | Grimsrud (2021) | This study was a review of findings on acceleration of differentiated service delivery for HIV treatment in sub-Saharan Africa during COVID-19, however, previously published review study was not stated as an inclusion criteria in the current study | Review studies |
| 6 | Meherali (2021) | This study was a review of findings on impact of the COVID-19 Pandemic on Adolescents’ Sexual and Reproductive Health in Low-and Middle-Income Countries, however, previously published review study was not stated as an inclusion criteria in the current study | Review studies |
| 7 | Harries (2021) | No information on barriers or facilitators to SRH services during COVID-19 in Africa was reported in the study | No SRH barriers or facilitators identified |
| 8 | Mambo (2020) | No information on barriers or facilitators to SRH services during COVID-19 in Africa was reported in the study | No SRH barriers or facilitators identified |
